# Is It a Good Idea to Cultivate Lucid Dreaming?

**DOI:** 10.3389/fpsyg.2019.02585

**Published:** 2019-11-15

**Authors:** Raphael Vallat, Perrine Marie Ruby

**Affiliations:** ^1^Department of Psychology, Center for Human Sleep Science, University of California, Berkeley, Berkeley, CA, United States; ^2^DYCOG Team, Lyon Neuroscience Research Center, CNRS UMR 5292 - INSERM U1028 - Lyon 1 University, Bron, France

**Keywords:** consciousness, sleep, health, lucid dreaming downsides, sleep alteration, emotional regulation, training, recreational

## Introduction

Lucid dreaming (LD) is the process of being aware that one is dreaming while dreaming. In some cases, the dreamer may even gain control over a part of the dream plot and scenery. The scientific investigation of LD (experience already mentioned in Antiquity) did not start before the nineteenth century *(de Saint-Denys*, *1867**)*, and the use of objective methods to study LD only emerged a few decades ago (e.g., LaBerge, 1979, 1980, 1988; LaBerge and Rheingold, 1991; Levitan and LaBerge, 1994). Recently, LD gained visibility: surveys showed that 1/4 of all participants (*N* = 1,380) had heard of LD, that LD research is no longer seen as esoteric, and that the public has a generally positive view on LD (Lüth et al., 2018; Neuhäusler et al., 2018).

With the emergence of a digital lifestyle in rich countries and hyper-realistic video games, it became obvious to an ever-increasing amount of people that LD is the ultimate form of immersive experience. Indeed, it offers a (free) unique and fantastic world in which everything may become possible or controllable and feels real without putting the dreamer at risk. These characteristics (fantastic sensory and emotional experience) make LD indubitably highly desirable (e.g., Stumbrys et al., 2014).

There is however a problem preventing most of the population from enjoying LD: spontaneous LD is not frequent. About 50% of individuals have experienced at least one lucid dream in their lifetime, and only 11% report having two or more lucid dreams per month (Schredl and Erlacher, 2011; Saunders et al., 2016; Vallat et al., 2018).

It is not surprising, in this context, that numerous training methods and devices aiming at increasing LD frequency and the level of control within the dream have been developed and commercialized in recent years. The various LD induction methods can be classified in three categories: (1) cognitive techniques, (2) external stimulation during sleep and, (3) intake of specific substances (Stumbrys et al., 2012; Dyck et al., 2017; Bazzari, 2018; LaBerge et al., 2018). Reviews highlighted that none of these induction techniques were verified to induce LD reliably and consistently. However, for lack of anything better, individuals who want to increase their LD frequency may use one of these methods.

## Sleep Disruption Risk Due to LD Induction Methods

Several of the LD induction methods deliberately (or incidentally) alter sleep architecture or duration. In the cognitive technique category, this is especially true of the widely-used mnemonic induction of lucid dreams technique (MILD; Levitan and LaBerge, 1994; Neuhäusler et al., 2018). The MILD is indeed more efficient if the trainee awakens during the night, stays awake for 30–120 min and then goes back to sleep (Stumbrys et al., 2012). This observation led to the development of the Wake-up-back-to-Bed technique, a LD induction method based solely on forced awakenings and periods of wake during the night. Those methods disturb sleep by increasing its fragmentation, modifying its architecture and decreasing its duration. Likewise, the dream re-entry method recommends counting while falling asleep after a short awakening, which may prevent trainees from actually falling asleep (Stumbrys et al., 2012).

Regarding the stimulation methods category, the principle is to deliver stimuli during sleep to trigger lucidity. Such stimulation is intrinsically associated with the risk of awakening (or arousing) the participants, and thus of decreasing sleep depth, disrupting sleep architecture and/or shortening sleep duration. The combination of the MILD techniques with external stimulation has also been tested because it was considered promising to induce LD (LaBerge, 1988; Levitan and LaBerge, 1994). In this case the risk of sleep disruption of the two techniques is cumulative.

Several substances have also been used to stimulate LD (via intracerebral acetylcholine increase), often in combination with the MILD technique (e.g., LaBerge et al., 2018; Baird et al., 2019). In this case, in addition to the previously mentioned risk, there is also the risk of disturbing the balance between the serotonergic and cholinergic systems which are jointly involved in regulating sleep. Disturbing this balance may impact sleep structure integrity (i.e., increased sleep fragmentation, time awake during the night, and sleep paralysis) and have adverse effects on health (Stumbrys et al., 2012; Biard et al., 2015, 2016).

Considering the gigantic amount of scientific evidence linking poor-quality or insufficient sleep to adverse health outcomes (including shorter life expectancy), and especially of sleep fragmentation in altered physical and cognitive health (e.g., Stepanski, 2002; Bonnet and Arand, 2003; Mullington et al., 2009; Mary et al., 2013; Walker, 2017, 2019; Ahuja et al., 2018; Barnes and Watson, 2019; Brauer et al., 2019; Pichard et al., 2019), one may seriously question the health consequences of regularly practicing LD induction methods.

## The Modified Cerebral State During LD

The experimental investigation of LD is challenging given the difficulty to get LD in the lab. Indeed, LD is rare and unpredictable even for frequent lucid dreamers, especially in an unfamiliar experimental setting. Nonetheless, by applying the method of LD objective detection (pre-determined ocular signaling, LaBerge and Rheingold, 1991) to EEG and fMRI, some determined neuroscientists have managed to get a glimpse of the cerebral correlates of LD. In a pioneering EEG study, Voss et al. (2009) succeeded in recording the brain activity of three dreamers while they were experiencing a lucid dream. They observed an increased activity in the gamma frequency band in the frontal lobe in lucid rapid eye movement (REM) sleep as compared to non-lucid REM sleep and concluded that LD constitutes a hybrid state of consciousness in-between sleep and wake (Hobson, 2009), with definable and measurable differences from waking and from REM sleep, particularly in frontal areas. This is coherent with the fact that most LD induction methods promote an increase of the arousal level during sleep, and suggest that anything susceptible to awaken the subject gradually, including nightmares, might favor or induce LD (e.g., Schredl and Erlacher, 2004). In line with this idea, a case fMRI study showed that lucid REM sleep was associated with a reactivation of areas that are normally deactivated during REM sleep, such as bilateral precuneus, parietal lobules, and prefrontal and occipito-temporal cortices (Dresler et al., 2012). These regions are involved in higher cognitive functions such as self-awareness and executive functions, and their reactivation during LD could account for the resurgence of a certain level of self-awareness and voluntary control (Hobson, 2009; Zink and Pietrowsky, 2015). In support to this hypothesis, an increased level of self-reflective awareness during dreaming was induced by fronto-temporal transcranial alternating current stimulation (tACS) (Bray, 2014; Voss et al., 2014). This study encouraged people to use tACS to induce LD, which again raises questions about safety notably of chronically using a method that affect cortical electrical activity (there are currently no clinical information on chronic or repeated use of tACS).

## Sleep Disruption Risk Due to an Increase of LD Frequency

In the case of a spontaneous increased LD frequency without any use of LD induction methods, one may still wonder what is the impact of “replacing” a regular sleep stage by a hybrid sleep stage on general health and notably on the function of sleep, given the well-known involvement of good sleep in good health and especially of REM sleep in emotional regulation and memory consolidation (e.g., Rauchs et al., 2005; Walker and van der Helm, 2009; Perogamvros and Schwartz, 2013; Plailly et al., 2019). Since there are now evidences that the brain is not functioning in the same way during lucid and non-lucid REM sleep (Voss et al., 2009, 2014; Dresler et al., 2012), one cannot exclude that an increase of lucid REM to the detriment of non-lucid REM may alter or diminish the outcome of regulation processes known to be at play during non-lucid sleep (Walker and van der Helm, 2009; Perogamvros and Schwartz, 2013; Ahuja et al., 2018; Lewis et al., 2018; Tempesta et al., 2018).

## Discussion

There are several reasons to fear an adverse effect on sleep and health of a regular use of LD induction methods or of an increased LD frequency, since (1) LD induction methods alter sleep integrity and (2) the brain state during LD is neither that of wake nor that of REM sleep, but rather a hybrid one that is naturally infrequent. Such concerns regarding the possible danger of LD training for sleep integrity are acknowledged on the web. On Google Search's top listing^1^ (at the time of writing) for “lucid dreaming,” one can read “*Another concern is that engaging in lucid dreaming requires focus and effort, which might mean that the sleeper does not get enough rest.”* Yet, such acknowledgment are mostly absent from the current scientific literature, and only a handful of studies have investigated the potential downsides of LD. The few existing experimental works are not visible and confirm the feared prediction by showing a significant relationship between LD frequency and poor sleep quality (Schadow et al., 2018; *N* = 1824). Similarly, Mota et al. (2016) showed that LD practice may further empower deliria and hallucinations in a psychotic population.

Our goal is therefore to draw attention to the fact that, as of today, we do not have a well-educated and clear idea of the consequence that training and cultivating LD may have on sleep integrity and more generally on health. This is even more important to highlight that there is a tendency in scientific and lay publications toward encouraging LD and not mentioning the possible side effects of LD training methods (e.g., Hobson, 2009; Mota-Rolim and Araujo, 2013; Stumbrys et al., 2016; Dyck et al., 2017). For example, Dyck et al. (2017) encourage to increase LD induction methods duration without mentioning possible adverse effect on sleep “*Future studies should extend the training period and increase participants' motivation by using social media technology in order to evaluate what techniques might be beneficial in a home setting for a group of participants not specifically selected for high interest in lucid dreaming*.” One can further read in Mota-Rolim and Araujo (2013): “*LD may allow for motor imagery during dreaming with possible improvement of physical rehabilitation*,” and in Stumbrys et al. (2016): “*Lucid dreaming practice provides a more realistic simulation of the waking environment than mental practice and could be alternatively used when an athlete is injured, unable to practice physically or actions are dangerous […] While only a limited number of athletes have lucid dreams on a frequent basis, there is a wide range of techniques that can be used for lucid dream induction*.” In these two latter publications LD is encouraged to achieve what could be done as effectively by motor imagery during wake (i.e., improved motor performance, as shown by the authors in Stumbrys et al., 2016), and without mentioning the possible side effects of LD practice on sleep. LD is also recommended in several publications (e.g., Mota-Rolim and Araujo, 2013; Morgenthaler et al., 2018; Sparrow et al., 2018) as a possible way to diminish nightmare frequency, even though several behavioral techniques preserving sleep are working very efficiently for this matter (e.g., Krakow and Zadra, 2006; Casement and Swanson, 2012; Putois et al., 2019; Imagery Rehearsal Therapy).

Our opinion is thus that one needs to be cautious and responsible regarding recommendations to practice LD training methods and a state (LD) whose consequences on health are unknown and understudied. To improve the safety of experimental use of LD in research or as a recreational activity, future studies would need to investigate the above-discussed downsides of LD induction methods practice and of LD frequency increase, and characterize them.

## Conclusion

In this opinion paper, we draw the attention to the possible adverse effect of LD on sleep and health. There are several reasons leading to fear that LD, and especially training to increase LD frequency, may be detrimental to normal sleep and notably to the sleep-related regulation processes. Our aim is to encourage future studies to recognize the lack of knowledge regarding possible side effects of LD induction methods or LD frequency increase, as well as to investigate such side effects to better characterize what they are and in which context they appear.

## Author Contributions

All authors listed have made a substantial, direct and intellectual contribution to the work, and approved it for publication.

### Conflict of Interest

The authors declare that the research was conducted in the absence of any commercial or financial relationships that could be construed as a potential conflict of interest.

## Footnotes

^1^https://www.medicalnewstoday.com/articles/323077.php#12

## Abbreviations

EEG: electroencephalography
fMRI: functional magnetic resonance imaging
LD: lucid dreaming.
